# On-Chip Training Spiking Neural Networks Using Approximated Backpropagation With Analog Synaptic Devices

**DOI:** 10.3389/fnins.2020.00423

**Published:** 2020-07-07

**Authors:** Dongseok Kwon, Suhwan Lim, Jong-Ho Bae, Sung-Tae Lee, Hyeongsu Kim, Young-Tak Seo, Seongbin Oh, Jangsaeng Kim, Kyuho Yeom, Byung-Gook Park, Jong-Ho Lee

**Affiliations:** Department of Electrical and Computer Engineering, Inter-University Semiconductor Research Center, Seoul National University, Seoul, South Korea

**Keywords:** neuromorphic, spiking neural networks, deep neural networks, on-chip training, supervised learning, hardware-based neural networks, synaptic devices

## Abstract

Hardware-based spiking neural networks (SNNs) inspired by a biological nervous system are regarded as an innovative computing system with very low power consumption and massively parallel operation. To train SNNs with supervision, we propose an efficient on-chip training scheme approximating backpropagation algorithm suitable for hardware implementation. We show that the accuracy of the proposed scheme for SNNs is close to that of conventional artificial neural networks (ANNs) by using the stochastic characteristics of neurons. In a hardware configuration, gated Schottky diodes (GSDs) are used as synaptic devices, which have a saturated current with respect to the input voltage. We design the SNN system by using the proposed on-chip training scheme with the GSDs, which can update their conductance in parallel to speed up the overall system. The performance of the on-chip training SNN system is validated through MNIST data set classification based on network size and total time step. The SNN systems achieve accuracy of 97.83% with 1 hidden layer and 98.44% with 4 hidden layers in fully connected neural networks. We then evaluate the effect of non-linearity and asymmetry of conductance response for long-term potentiation (LTP) and long-term depression (LTD) on the performance of the on-chip training SNN system. In addition, the impact of device variations on the performance of the on-chip training SNN system is evaluated.

## Introduction

Recently, artificial neural networks (ANNs) have shown superior performance in several fields, such as pattern recognition or object detection (Gokmen and Vlasov, 2016; Ambrogio et al., 2018; Kim C.-H. et al., 2018; Kim J. et al., 2018; Kim et al., 2019). The structure of ANNs was inspired by models of cortical hierarchies in neuroscience and neuroengineering (Fukushima, 1988; Riesenhuber and Poggio, 1999; Pfeiffer and Pfeil, 2018). In particular, convolutional neural networks (CNNs) inspired by the biological vision model have significantly improved the accuracy of deep neural networks (Krizhevsky et al., 2012). However, it is difficult to say that the ANNs with a Von Neumann architecture perfectly imitate a human’s brain, which is a very high-speed and massively parallel operating system with ultra-low power consumption (O’Connor et al., 2013; Shrestha et al., 2018; Kang et al., 2019). In light of this, hardware-based spiking neural networks (SNNs) capable of massively parallel operation by using analog synaptic devices have been regarded as an innovative type of computing system that can partially replace ANNs (Hwang et al., 2018).

Spiking neural networks can imitate biological behavior with various neuron and synapse models (Jo et al., 2010; Yang et al., 2016). Neurons in SNNs generate spikes to communicate between adjacent neurons. The input intensity of the neuron is represented as the number of spikes generated from the neurons (Oh et al., 2019). The spikes transmit through synapses and are integrated into the membrane capacitor of neurons in the next layer. When the membrane potential exceeds the threshold voltage, the neuron generates a spike to the deeper layer. This biological behavior of the neuron in SNNs can be matched to the behavior of the rectified linear unit (ReLU) activation function in ANNs (Diehl et al., 2015; Rueckauer et al., 2017). Since their behavior can be matched with each other, weights trained in ANNs with ReLU can be exactly converted to the weights in SNNs with very slight accuracy degradation. Using the ANN-to-SNN conversion method, SNNs have achieved state-of-the-art accuracy in MNIST, CIFAR-10, and Imagenet classification (Pfeiffer and Pfeil, 2018). However, the weights in SNNs should be trained from ANNs in serial operation, and the conversion is performed once. Therefore, SNNs adopting the ANN-to-SNN conversion cannot update themselves depending on various system situations and only perform the inference process for a given task. For this reason, the performance of SNNs that adopt conversion is sensitive to unexpected variations of hardware and cannot save the power consumption required for training a weight (Kim H. et al., 2018; Yu, 2018). In contrast, SNNs using on-chip training schemes that can update weights on the chip can have immunity against device variation or noise (Querlioz et al., 2013; Kwon et al., 2019). In addition, the on-chip training SNN systems train a weight by applying an update pulse to a synaptic device representing a weight, which leads to low power consumption for training a weight (Hasan et al., 2017).

There are two types of training weight methods for SNNs on the chip. One imitates the unsupervised training behavior in the human brain, for example, spike-timing-dependent plasticity (STDP) algorithms (Bi and Poo, 1998; Milo et al., 2016; Kheradpisheh et al., 2018). The other type is the supervised training method, which updates weights by approximating the backpropagation algorithm to match the behavior of the SNNs (Lee et al., 2016; Tavanaei and Maida, 2019). SNNs using unsupervised STDP have been reported to be implemented with synaptic devices, such as RRAM or Flash devices (Pedretti et al., 2017; Kim C.-H. et al., 2018; Prezioso et al., 2018). However, compared to conventional ANNs, the performance of SNNs using STDP is limited in terms of accuracy. In contrast to STDP, the performance of SNNs using approximated backpropagation is close to that of conventional ANNs. However, even in this case, signals representing an error value should be propagated backward while calculating and storing the values for updating weights, which is the main reason why it is difficult to implement hardware-based SNNs using on-chip training schemes.

Here, we propose a new supervised on-chip training scheme that efficiently approximates the backpropagation algorithm suitable for SNNs. The proposed on-chip training scheme dramatically reduces the memory usage required for the weight update by using 1 bit of memory per neuron to determine whether the neuron generates a spike at the last time step, and 1 bit of memory per neuron to store the derivative of the neuron’s activation function. By using the stochastic characteristic of neurons in SNNs, the performance of SNNs using the proposed training scheme achieves the performance of ANNs. For the hardware configuration of on-chip training SNN systems, a gated Schottky diode (GSD), which has a saturated current, is fabricated as a synaptic device (Bae et al., 2017; Lim et al., 2019b). This characteristic greatly improves the reliability of the SNN system by allowing the GSDs to represent accurate weights even if an unexpected voltage drop occurs in the system (Lim et al., 2019a). In addition, a parallel conductance update scheme that speeds up the SNN system is validated for GSDs. We then design and simulate an on-chip training SNN system based on the results measured from GSDs and verify the performance of the system based on its ability to classify MNIST data sets. Lastly, the system is evaluated for non-ideal characteristics of synaptic devices, such as non-linearity, asymmetry, and device variation.

## Materials and Methods

### Gated Schottky Diode

**FIGURE 1:**
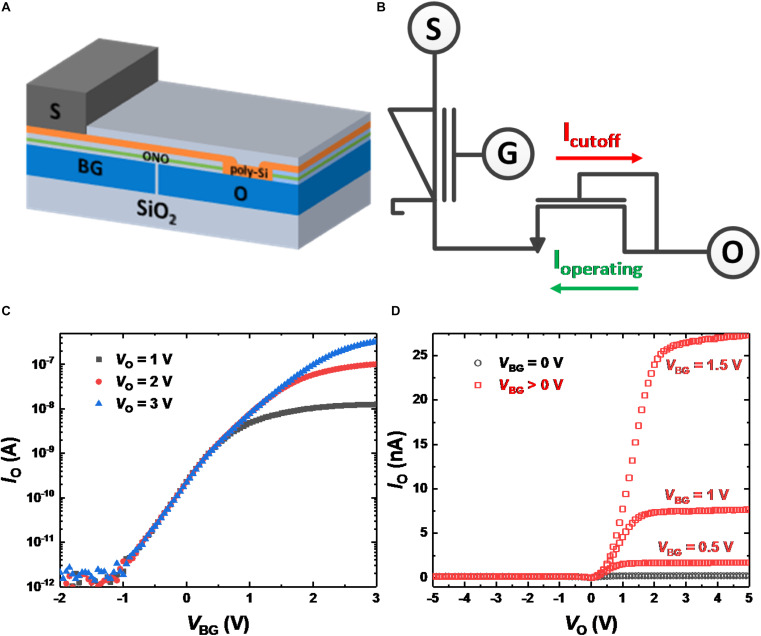
**(A)** Bird’s eye view of a GSD. **(B)** Circuit diagram of a GSD. When *V*_BG_ is positive, operating current flows with positive *V*_O_, and the current is cut off with negative *V*_O_. **(C)** Measured *I*_O_-*V*_BG_ curves of GSDs with different *V*_O_ values. **(D)** *I*_O_-*V*_O_ curves of GSDs with different *V*_BG_ values. When *V*_O_ is positive, saturated current is shown as *V*_O_ increases. Since a negative *V*_O_ depletes electrons in the poly-Si active layer, the *I*_O_ current is cut off.

A three-terminal gated Schottky diode (GSD) that cuts off the Schottky forward current was previously fabricated to act as a synaptic device (Lim et al., 2019b). However, the GSD in the previous paper was damaged by the sputtering process for the deposition of metal electrodes. By reducing the sputtering power, the current level of the GSD is improved. Figure 1A shows a bird’s eye view of the GSD. The bottom gate (BG) and ohmic contact (O) are made of *n*^+^-poly silicon. A SiO_2_/Si_3_N_4_/SiO_2_ (ONO) stack is then deposited, and the Si_3_N_4_ layer acts as a charge storage layer. As an active layer, undoped Si is deposited on the ONO stack and O electrode. Contact holes are opened on the active layer after the layer of SiO_2_ has formed. A Ti/TiN/Al/TiN stack is deposited on the exposed active layer by sputtering and forms the Schottky contact (S). Figure 1B shows a circuit diagram of an *n*-type GSD when the voltage applied to the BG is positive. If *V*_BG_ is positive, the Schottky junction is formed at the S contact, and NMOS is formed intrinsically within the structure of the GSD. Measured *I*_O_-*V*_BG_ curves of GSDs for different *V*_O_ values are shown in Figure 1C. The effective Schottky barrier height for electrons decreases as *V*_BG_ increases, and the operating current of GSD is the reverse Schottky diode current. Therefore, the reverse Schottky diode current also increases as *V*_BG_ increases and can be used as a weight for SNNs. In addition, since the magnitude of the reverse Schottky diode current is low, an SNN system using GSDs operates with low power consumption. Figure 1D shows the measured *I*_O_-*V*_O_ curves of GSDs with different *V*_BG_ values. Since *V*_O_ above a certain value (e.g., 1.5 V at *V*_BG_ = 1 V) is dropped between O and S in Figure 1B, the reverse Schottky diode current is saturated with respect to the input voltage of *V*_O_. With the help of the saturation behavior, the current of a GSD does not change despite voltage drops along metal wires in a crossbar array, and voltage drops by electronic switches do not affect the voltage across the device (Lim et al., 2019a). In addition, negative *V*_O_ depletes electrons in the Si active layer when *V*_BG_ is positive, and Schottky forward current is blocked.

**FIGURE 2:**
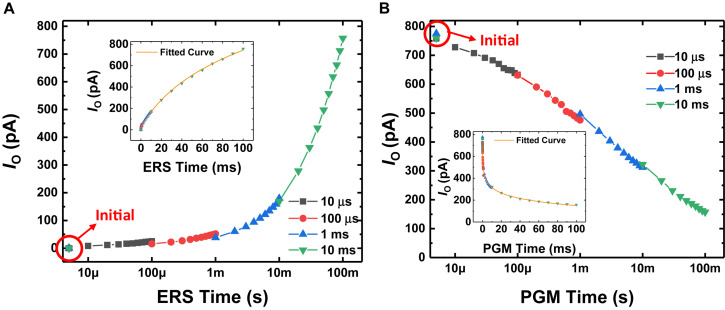
*I*_O_ behavior with respect to **(A)** erase time and **(B)** program time when *V*_O_ is 3 V and *V*_BG_ is 0 V. After the GSD is initialized, erase (–7 V) or program (5.5 V) pulses are applied to the BG electrode, with 0 V applied to the S and O electrodes. Each pulse with a different pulse width is applied to the GSD 10 times. (Inset) *I*_O_ behavior with respect to the erase or program time on a linear scale.

Figures 2A,B show the conductance response (*I*_O_ at *V*_O_ = 3 V, *V*_BG_ = 0 V, *V*_S_ = 0 V) with respect to the time the erase pulse (*V*_BG_ = −7 V, *V*_O_ = 0 V, *V*_S_ = 0 V) and program pulse (*V*_BG_ = 5.5 V, *V*_O_ = 0 V, *V*_S_ = 0 V) are applied, respectively. Long-term potentiation (LTP) and long-term depression (LTD) curves are shown by applying the erase and program pulses, respectively. After GSDs are initialized, each pulse with a different pulse width is applied to the GSDs 10 times. Since the amount of charge stored in the Si_3_N_4_ layer is determined by the total time the FN tunneling current flows (Kim et al., 2010), the conductance can be changed continuously with the time of the program or erase pulses applied to the devices. The normalized conductance response of the GSD is fitted by the model of conductance with respect to the total time a pulse is applied to a synaptic device (Querlioz et al., 2011; Ernoult et al., 2019; Kwon et al., 2019), as follows:

(1) $$G_{L\,T\,P}\,\left(t\right)=a_{L\,T\,P}+\frac{1}{\beta_{L\,T\,P}}\,\ln\left(t+c_{L\,T\,P}\right),f\,o\,r\,L\,T\,P$$

(2) $$G_{L\,T\,D}\,\left(t\right)=a_{L\,T\,D}-\frac{1}{\beta_{L\,T\,D}}\,\ln\left(t+c_{L\,T\,D}\right),f\,o\,r\,L\,T\,D$$

where *G* is the conductance of the synaptic device, *t* is the total time the pulse is applied, *a* and *c* are the fitting parameters, and β is a non-linearity factor. As shown in Figure 2, the GSDs have a near-linear LTP curve (β_*LTP*_ of ∼1.60) and a non-linear LTD curve (β_*LTD*_ of ∼8.03). The normalized conductance responses as a parameter of the non-linearity factor are described in Supplementary Figure S1.

### On-Chip Training Algorithm

The behavior of an integrate-and-fire (I&F) neuron in an SNN can approximate the conventional ReLU activation function in ANNs (Tavanaei and Maida, 2019). A ReLU activation function, *f*(*y*), is defined as follows:

(3) f⁢(y)=max⁡(0,y),

(4) $$\frac{d\,f}{d\,y}=\left\{ \begin{array}{c} 1,y>0\\ 0,y\leq 0 \end{array}\right.$$

where *y* is the input signal of the activation function. When the input signal of ReLU exceeds 0, the activated value is propagated to the next layer, and the derivative of ReLU is set to 1. This behavior of ReLU is similar to the behavior of I&F neurons, which also generate and propagate a spike when the membrane potential exceeds the threshold voltage. In this regard, I&F neurons are used in the forward-propagation phase (FP), the phase for the inference process. In addition, we approximate the derivative of the activation function of I&F neurons in the form of a derivative of ReLU.

**FIGURE 3:**
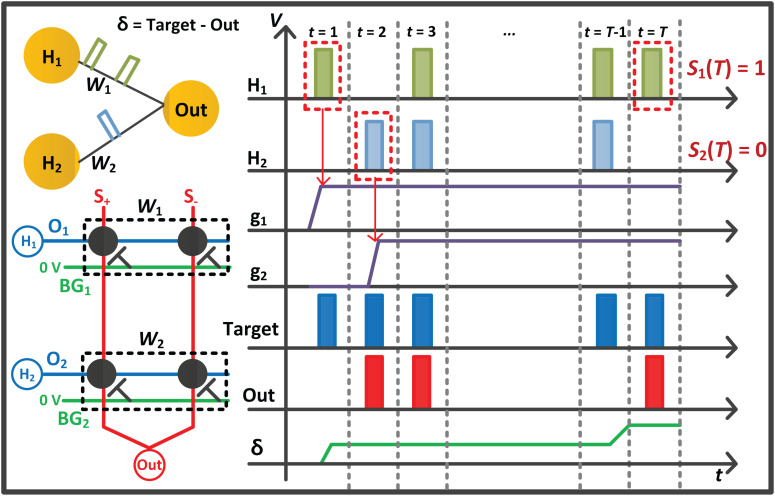
Conceptual diagram with a 1-layer network for the forward phase (FP) of the proposed on-chip training scheme. The spikes from previous layers propagate along the O line of the *G*^+^ and *G*^–^ array, and the current sum of the array is integrated into the membrane capacitor of the I&F. When the neuron H_1_ and H_2_ generate a spike, the derivative (g_1_ and g_2_) of the neurons is set to a value of 1.

In SNNs, a weight is represented by the conductance difference between two synaptic devices representing positive and negative values. In the case of a network having *L* layers, a weight connecting neuron *i* in layer *l* to neuron *j* in layer *l* + 1 is represented by $W_{i\,j}^{l}=G_{+,i\,j}^{l}-G_{-,i\,j}^{l}$, where **l* ∈ {1, …, *L*−1}* (Burr et al., 2015). The input of the first layer is converted to a Poisson-distributed spike train, and the input intensity is encoded as a spike rate. The input spikes are fed into the GSD arrays, which represent the weight matrix. An I&F neuron integrates charge resulting from the weighted sum into its membrane capacitor:

(5) $$V_{j}^{l}\,\left(t_{F\,P}\right)=V_{j}^{l}\,\left(t_{F\,P}-1\right)+\sum_{i}^{N^{l-1}}\frac{S_{i}^{l-1}\,\left(t_{F\,P}\right)}{C_{m\,e\,m}}\,\left(G_{+,i\,j}^{l-1}-G_{-,i\,j}^{l-1}\right),$$

where *V^*l*^_j_*(*t*_FP_) is the membrane potential of I&F neuron *j* in layer *l* at time step *t*_FP_, *N*^*l*–1^ is the total number of neurons in layer *l*-1, *S^*l–*1^_i_*(*t*_FP_) is a spike in the form of a voltage pulse generated from neuron *i* in layer *l*-1 at time *t*_FP_, and *C*_mem_ is the membrane capacitance of an I&F neuron. The voltage pulses propagate along the O lines in the GSD array, and the currents along the O lines are added to the S lines in the array. The current output from the GSD array charges or discharges the membrane capacitor of an I&F neuron. The I&F neuron generates a spike when its membrane potential exceeds the firing threshold voltage of the I&F neuron (*V*_th_). *V*_th_ is then subtracted from the membrane potential of the neuron_:_

(6) $$i\,f\;V_{j}^{l}\,\left(t_{F\,P}\right)>V_{t\,h}:\left\{ \begin{array}{c} V_{j}^{l}\,\left(t_{F\,P}\right)=V_{j}^{l}\,\left(t_{F\,P}\right)-V_{t\,h}\\ S_{j}^{l}\,\left(t_{F\,P}\right)=1\\ g_{j}^{l}=1 \end{array}\right.$$

(7) $$e\,l\,s\,e:S_{j}^{l}\,\left(t_{F\,P}\right)=0,$$

where *g* is an approximated derivative of the neuron’s activation function. When FP starts for a given input signal, the approximated derivative *g* of each neuron’s activation function is initialized to 0. Then, if the neuron generates a spike during FP, *g* is set to 1. If the neuron does not generate a spike during FP, g remains 0. Although the behavior of an I&F neuron cannot be differentiable, neural networks have been reported to show comparable performance when storing a derivative with only 1 bit (Narayanan et al., 2017; Tavanaei and Maida, 2019). In the last layer (*l* = *L*), spikes generated from the neurons and target spikes that supervise the correct answer are accumulated to obtain the “delta” value in the last layer (δ^*L*^):

(8) $$\delta_{j}^{L}=k\,\sum_{t_{F\,P=1}}^{T}\left(T\,a\,r\,g\,e\,t_{j}\,\left(t_{F\,P}\right)-S_{j}^{L}\,\left(t_{F\,P}\right)\right),$$

where *T* is the total time step for the FP, and *k* is a constant that converts the number of spikes into the voltage amplitude. For the correct label, a target spike train has a value of 1, and its firing frequency is set to the maximum. In other words, a target spike is generated every time step with the value of 1 for the correct label, and no target spike is generated for the wrong label. The constant *k* is set to the value with the maximum $\delta_{j}^{L}$ of 1 V. The whole process performed in the FP is simply described with a 1-layer network in Figure 3.

In the backward-propagation phase (BP), the delta values reversely propagate to the previous layer through the synaptic devices and are integrated to obtain the delta sum (Burr et al., 2015; Hasan et al., 2017; Narayanan et al., 2017; Ambrogio et al., 2018):

(9) $$\delta_{i}^{l}=\sum_{j}^{N^{l+1}}\frac{\lambda_{B\,P}\,\delta_{j}^{l+1}}{C_{B\,P}}\,\left(G_{+,i\,j}^{l}-G_{-,i\,j}^{l}\right)\,g_{i}^{l},$$

where *g* is the derivative of the neuron’s activation function determined in the FP. λ is a constant representing the ratio of voltage pulse width to voltage amplitude, and *C*_BP_ is the capacitance to store δ. The δ is obtained in the form of voltage amplitude and is converted to a voltage pulse (λδ) with a width proportional to the voltage amplitude using the pulse-width modulation circuit (Hasan et al., 2017; Lim et al., 2019a). Although the current direction of GSDs in the BP should be kept the same as in the FP to maintain their conductance value, the delta sum can be performed along the O line of GSD arrays while maintaining the current flow direction (Lim et al., 2018). Then, $\delta_{i}^{l}$is obtained when the corresponding derivative ($g_{i}^{l}$) determined in the FP is 1.

In the update phase (UP), the conductance of synaptic devices is updated depending on δ. In the conventional backpropagation algorithm, the weight ($W_{i\,j}^{l}$) update is calculated as $\text{Δ}\,W_{i\,j}^{l}\propto x_{i}^{l}\,\delta_{j}^{l+1}$, where $x_{i}^{l}$ is the activated value. When this update rule is applied to the SNNs, $x_{i}^{l}$ is matched to the number of spikes generated from the neuron during the FP. However, significant power consumption and memory usage are required for counting and storing the number of spikes for every neuron, which can become a bottleneck for the entire SNN system (Yu, 2018). In this work, we use a 1-bit spike value (0 or 1) per neuron depending on whether the neuron generated a spike at the last time step:

(10) $$\Delta \,t_{+,i\,j}^{l}=\Delta \,t_{-,i\,j}^{l}=\left|S_{i}^{l}\,\left(T\right)\times \lambda_{U\,P}\,\delta_{j}^{l+1}\right|,$$

where λ is a constant representing the ratio of voltage pulse width to voltage amplitude and Δ*t* is the width of the voltage pulse applied to the corresponding synaptic device. In the UP, since the amount of conductance update is modulated by λ, λ represents the learning rate of conventional ANNs. Whether it is a program pulse or an erase pulse is determined by the sign of the delta value. When the weight increases, the conductance of the synaptic device representing the positive weight increases by the erase pulse and the conductance of the synaptic device representing the negative weight decreases by the program pulse. On the contrary, when the weight decreases, the program pulse is applied to the device representing the positive weight and the erase pulse is applied to the device representing the negative weight. The whole training process of the proposed scheme is represented in Algorithm 1.

**Algorithm 1:** On-chip training scheme in SNNs with synaptic devices.

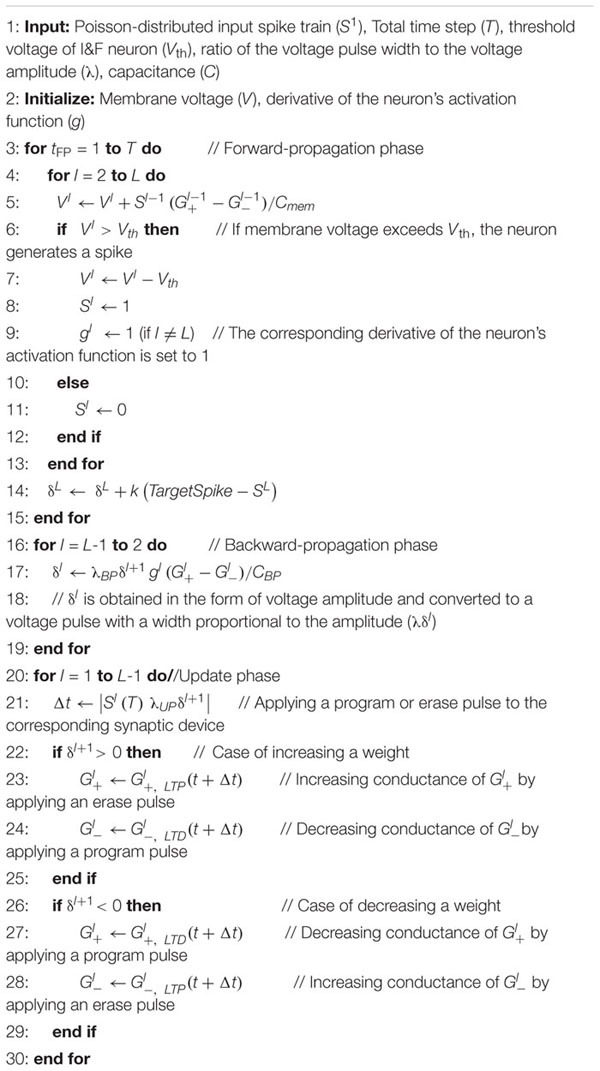

### Updating Method

**FIGURE 4:**
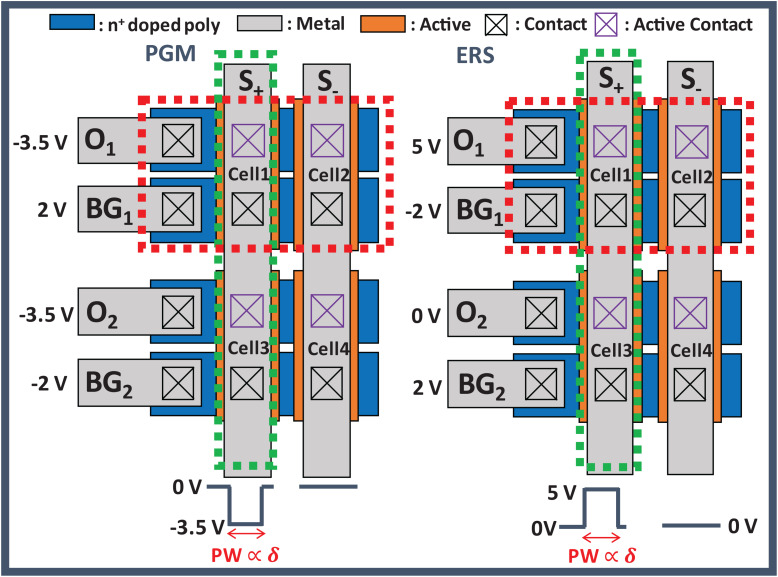
Bias condition in the update phase with the GSD array. The red dotted square is the condition along the BG and O line for *S*(T) of 1, and the green dotted square along the S line is the condition when δ is not equal to 0.

**FIGURE 5:**
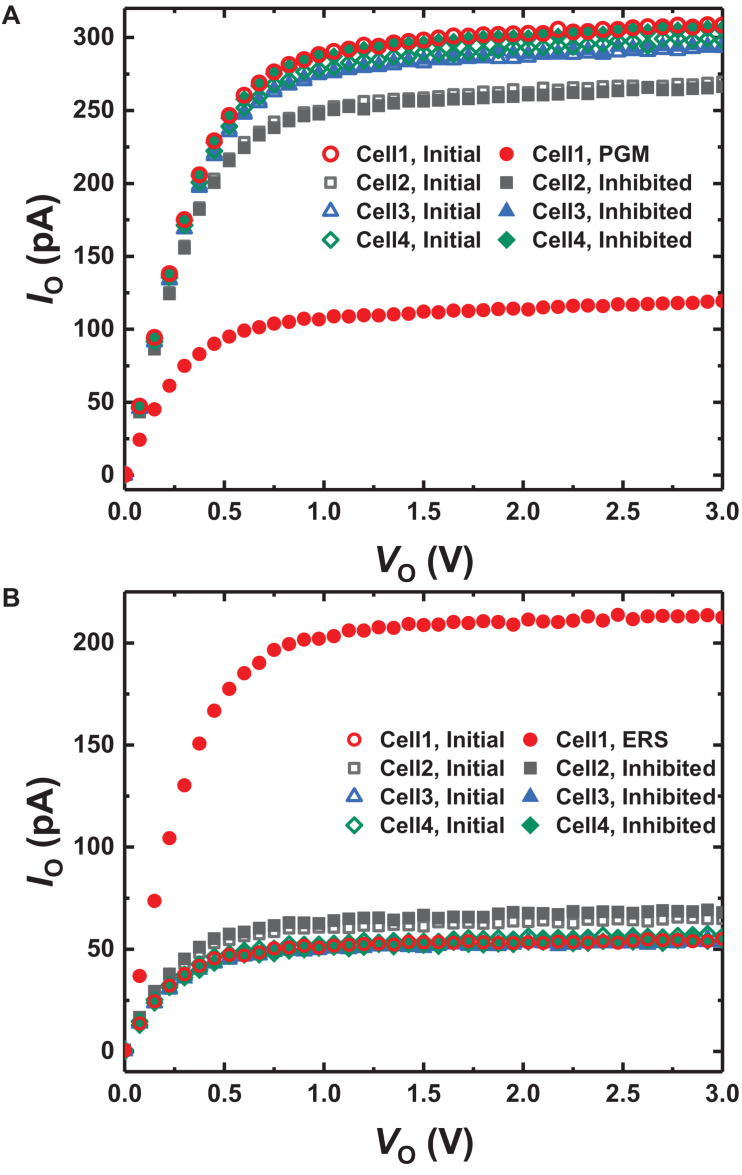
*I*_O_-*V*_O_ curves of the GSDs in the array **(A)** programming for 10 ms and **(B)** erasing for 100 ms depending on each condition in Figure 4. Only the current of cell 1 is changed, while others are inhibited successfully.

After all delta values (δ) except in the first input layer have been obtained, the conductance of the GSDs is updated by δ and *S*(*T*). To update the conductance of GSDs in parallel, we apply DC bias to the BG and O lines of the array and a program or erase pulse to the S lines of the array. Figure 4 shows the 2-by-2 layout of GSD arrays and the bias conditions of program and erase in the UP. The red dotted square represents the condition along the BG and O lines for *S*(*T*) of 1, and the green dotted square stands for the condition along the S line if δ is not equal to 0. The width of the program and erase pulses is proportional to δ, which can be implemented by the pulse-width modulation circuit (Lim et al., 2019a). In this case, only cell 1 in Figure 4 should be updated by a program or erase pulse, and the others should be inhibited in this condition. When a program pulse with an amplitude of −3.5 V is applied to the S line, the voltage of 2 V is applied to the BG line of cell 1. The voltage difference between the BG and S in cell 1 is then 5.5 V, which is the condition for programming a GSD. On the contrary, the voltage difference between BG and S of the other cells does not exceed 5.5 V, so the other cells are inhibited in this program scheme. In case of applying an erase pulse to the S line, the erase pulse has the same width as the program pulse width, but it has an amplitude of 5 V. The conductance change of each cell condition is shown in Figure 5. The width of the program pulse is 10 ms, and the width of the erase pulse is 100 ms. In both the cases of program and of erase, only the conductance of cell 1 is updated, and the others are inhibited successfully. By using this scheme, the GSDs in the array can be updated in parallel, which can improve the update speed of the entire SNN system. Note that the on-chip training SNN system updates weights as frequently as the training iterations, so a parallel conductance update of the device array is required to boost the training speed of the system.

## Results

### Evaluation of On-Chip Training Scheme

**FIGURE 6:**
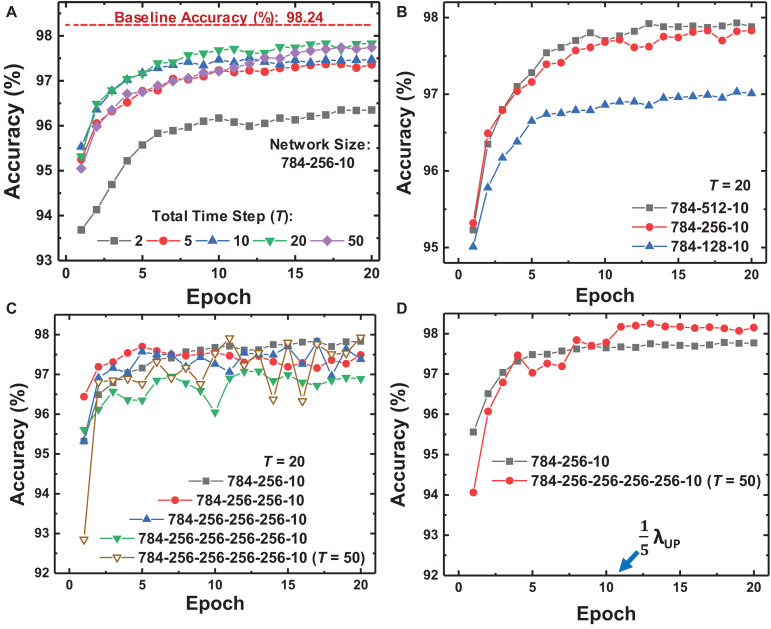
Training curves of the proposed on-chip training scheme depending on **(A)** the total time step (*T*) in FP, **(B)** the width of the hidden layer, and **(C)** the number of hidden layers. **(D)** Training curves when modulating λ at epoch 11. Deep networks show variance over epochs with large λ, but modulating λ stabilizes the training curve and improves the accuracy of the network.

We design and simulate fully connected (FC) neural networks for MNIST classification to verify the proposed on-chip training scheme for SNNs. The batch size of training is 1 to reduce memory usage and the area footprint required for the memory. The accuracy of SNNs is evaluated with the membrane voltage of the neuron at the last layer. The parameters in the training scheme for MNIST classification are described in Table 1. Figure 6A shows the MNIST test set accuracy of SNNs using the proposed on-chip training scheme according to the total time step (*T*). Here we assume that synaptic devices have a linear conductance response and no variation, and the baseline accuracy in Figure 6A is evaluated in ANNs that have the same network size. If *T* is 20, the maximum number of input, hidden, and output spikes are 20. The increased *T* precisely represents the activation value of each neuron and δ, resulting in improved accuracy for SNNs. When *T* is equal or more than 20, the SNNs show saturated accuracy but achieve accuracy near the baseline accuracy of ANNs. Figures 6B,C show whether the proposed on-chip training scheme can be applied to wider and deeper networks. The on-chip training SNNs achieve higher accuracy as the layer width increases, but the accuracy decreases as the depth of the network increases with the same *T*. In this case, since increased *T* represents more accurate neuron activation values and δ, the accuracy in deeper networks is expected to be improved. As a result of increasing *T* to 50, the accuracy of SNNs with 4 hidden layers increases, as shown in Figure 6C. Nevertheless, the training curve for the network with 4 hidden layers oscillates over epochs due to the large λ_UP_. Since λ_UP_ is multiplied by δ, a large λ_UP_ increases the amount of weight update and causes the oscillating training curve. Thus, we scale λ_UP_ to train deeper networks. After reducing λ_UP_ to 0.2λ_UP_ at epoch 11 in Figure 6D, a stable training curve is obtained, and the accuracy increases to 98.25%.

**TABLE 1:** The parameters when the GSDs are used as synaptic devices in the SNNs.

**Parameters**	**Description**	**Value**
*a*_LTP_, *a*_LTD_ *c*_LTP_, *c*_LTD_	Parameters of the fitted curve for the normalized conductance response of GSDs	2.270, 1.422 0.0278, 18.25
β_LTP_,β_LTD_	Non-linearity factor of GSDs	1.60, 8.03
*S*	Spike in the form of voltage pulse	Pulse amplitude: 3 V Pulse duration: 10 μs
*C*_mem_	Membrane capacitance of I&F neuron	4*T*−8*T* fF (*l* = 1) 40−80 fF (*l* > 1)
*V*_th_	Threshold voltage of I&F neuron	0.1 V
λ	Ratio of voltage pulse width to voltage amplitude	BP 50 μs/V
		UP 50 μs/V (*l* = *L*) 500 μs/V (*l* ≠ *L*)
*C*_BP_	Capacitance for BP	40 fF

Table 2 compares this work with conventional on-chip training schemes using analog synaptic devices for MNIST classification. The proposed on-chip training scheme achieves an accuracy near that of conventional ANNs even when the batch size of training is 1 with a single hidden layer. In addition, we increase the batch size to 100 to improve the proposed scheme for SNNs with 4 hidden layers. Although increasing batch size for training directly increases memory usage, it improves the accuracy of deep networks. As a result, the network achieves an accuracy of 98.44% (0.1% lower than the accuracy of an ANN using the Adam optimizer), and shows excellent performance compared to other on-chip training schemes.

**TABLE 2:** Comparison of the proposed with conventional on-chip training schemes for hardware-based neural networks using analog synaptic devices.

**Architecture**	**Training method**	**Batch size**	**Network size**	**Accuracy**
FC (Zhang et al., 2018)	Sign BP	–	784-300-10	94.50%
FC (Chang et al., 2018)	BP-based	10–50	784-300-10	97.93%
FC (Fu et al., 2019)	BP-based	–	400-100-10	95.55%
FC (Ambrogio et al., 2018)	Adam	1	528-250-125-10	97.94%
FC (Lim et al., 2018)	Manhattan learning rule	1	784-200-10	95.36%
FC (ANN*)	Adam	100	784-256-256-256-256-10	98.54%
FC (This work)	BP-based	1	784-256-10	97.83%
FC (This work)	BP-based	100	784-256-256-256-256-10	98.44%

The accuracy of all reported data was evaluated through the classification of the MNIST test set and obtained through hardware-based simulation. The proposed on-chip training scheme shows excellent accuracy compared to other on-chip training schemes. * Software-based neural network simulated in the Pytorch framework.

**FIGURE 7:**
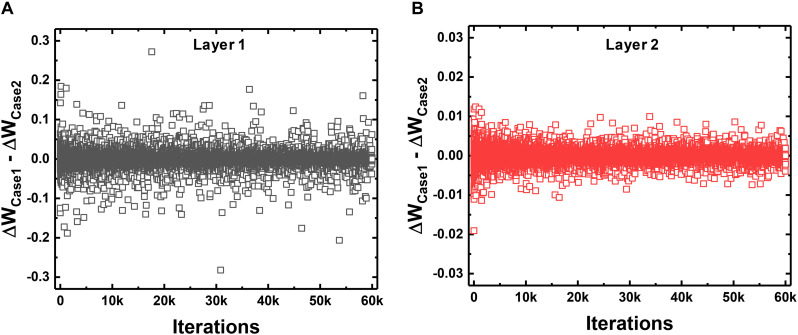
Comparison between the updating method that uses only a 1-bit spike event at the last time step per neuron (Case 1) and the total number of generated spikes in the neuron divided by the total time step (Case 2). The difference in the sum of total weight updates for Case 1 and Case 2 with respect to the training iterations in **(A)** the first layer and **(B)** the second layer.

**FIGURE 8:**
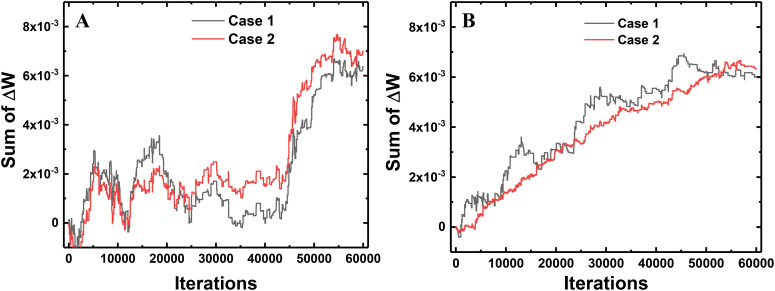
Sum of weight updates in a synapse connecting two adjacent neurons in the 784-256-10 network. **(A)** Sum of weight updates in the synapse between the 358th neuron as the input layer and the 124th neuron as the hidden layer. **(B)** Sum of weight updates in the synapse connecting the 97th neuron as the hidden layer and the 5th neuron as the output layer.

When ANNs are converted to SNNs, I&F neurons generate spikes at each time step with a probability proportional to the activated value in the ANN. Then, the weights connected to the neuron that generates a large number of spikes are updated with a high probability in one training iteration. This weight update scheme using a 1-bit spike event of a neuron is less accurate than that using the total number of spikes of a neuron. However, the average of total weight updates using a 1-bit spike approximates the average of total weight updates using the number of spikes of the neuron. To compare the weight update schemes, we trace the sum of total weight updates in each layer with respect to the training iterations. Case 1 is the sum of total weight updates using 1-bit spike events (this work), and Case 2 is the sum using the total number of spike events. In Case 1, *S*(*T*) of the equation (10) is 0 or 1, determined by the spike event at the last time step. In Case 2, *S*(*T*) in the equation (10) is converted to the number of spikes in the FP divided by *T*. For example, if the neuron generates spikes 14 times in the FP with a *T* of 20, the *S*(*T*) in the equation (10) is converted to 0.7 for Case 2. The actual weight update is performed with the 1-bit spike of a neuron, but the amount of the weight update is calculated by both ways at each iteration to compare them. Figure 7 shows the difference between the sum of total weight updates for Case 1 and Case 2. As shown in Figure 7, the sums of total weight updates in both cases are not exactly the same, but the values in Case 1 fluctuate around the values in Case 2. In addition, we trace the sum of weight updates of the random position in each layer: a synapse connecting the 358th neuron as the input layer and the 124th neuron as the hidden layer, and a synapse connecting the 97th neuron as the hidden layer and the 5th neuron as the output layer. As shown in Figure 8, the sum of weight updates in case 1 follows the curve for case 2, although the curves are not exactly the same. This indicates that the proposed on-chip training scheme for SNNs can achieve performance similar to that of ANNs by using the stochastic characteristics of SNNs. In other words, a spike from a neuron is generated at every time step with a probability proportional to the value of the neuron’s activation function, so the 1-bit spike event approximates the behavior of the neuron’s activation function during training.

### Non-ideal Device Characteristics

**FIGURE 9:**
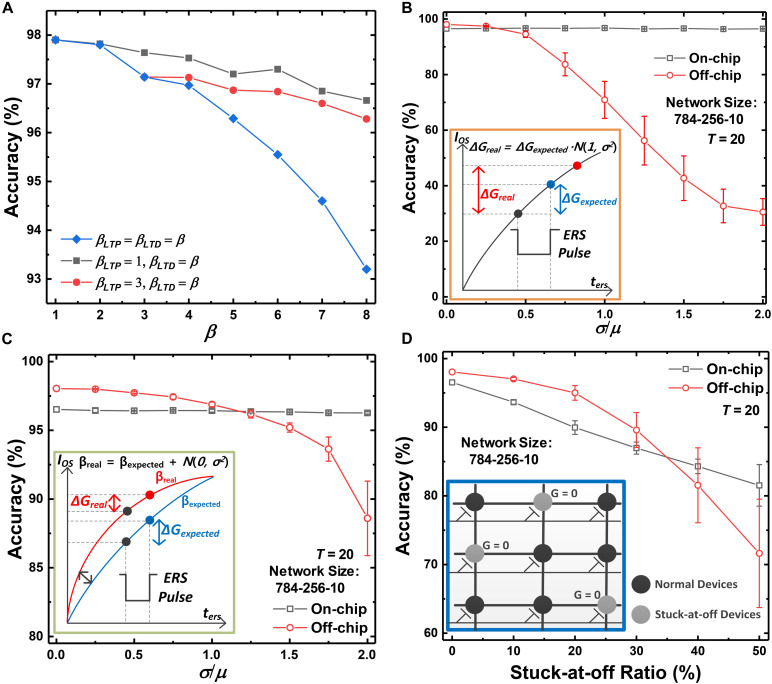
Accuracy of the on-chip training SNN systems versus **(A)** the non-linearity factor (β) of conductance response, **(B)** pulse-to-pulse variation, **(C)** device-to-device variation, and **(D)** stuck-at-off ratio. Although extremely non-linear and asymmetric devices are used as synaptic devices, high accuracy is obtained. Degradation due to pulse-to-pulse variation and device-to-device variation is negligible, but degradation due to the stuck-at-off ratio is significant.

The accuracy of on-chip training SNNs versus the non-linearity of conductance response is shown in Figure 9A. Although the delta value (δ) can be applied to the synaptic devices in the form of the program or erase pulse, the conductance response is non-linear with respect to the updating pulse. As a result, the expected weight updates cannot be reflected in the conductance updates, which causes accuracy degradation of SNNs. Nevertheless, an accuracy of higher than 93% is obtained when the non-linearity factor (β) is 8 for both LTP and LTD, which is an extremely non-linear conductance response of synaptic devices. Since the conductance of synaptic devices is updated continuously with the program or erase time, the on-chip training SNN system can achieve high accuracy even with highly non-linear devices. The accuracy of SNNs depending on the non-linearity for LTD is shown in Figure 9A to investigate the effect of asymmetry between the LTP and LTD curves on the accuracy. The non-linearity factor of the LTP curve has fixed values of 1 and 3. The accuracy of SNNs decreases as the non-linearity factor for LTD increases, represented as the red and black lines in Figure 9A. However, the degree of accuracy reduction resulting from the asymmetry is less than when β values for both LTP and LTD increase. In the case of a GSD as a synaptic device, the on-chip training SNN achieves an accuracy of 96.5%. The near-linear conductance change in the LTP curve can mitigate the effect of non-linear conductance change in LTD.

Inherent device variation is inevitable in neurons and synaptic devices. We categorize the device variation into three types: pulse-to-pulse variation (Chen et al., 2015), device-to-device variation (Gong et al., 2018; Sun and Yu, 2019), and stuck-at-off variation (Li et al., 2018). The performance of the proposed on-chip training scheme is evaluated with the degree of each variation and is compared with the performance of the off-chip training scheme (Kwon et al., 2019). When the off-chip training scheme is adopted to SNNs, the weights trained in ANNs using ReLU are exactly converted to the weights in SNNs by modulating the width of pulses applied to the synaptic devices. The synaptic device used in the SNNs is the GSD device, which has β values of ∼1.60 and ∼8.03 for LTP and LTD. In the off-chip training scheme, the trained weights are transferred to conductance along the LTP curve. When the ANN-to-SNN conversion is adopted, the accuracy of off-chip training SNNs with a *T* of 20 is 98.04% for MNIST data classification as a baseline. All accuracy datapoints in Figures 9B–D were evaluated five times and then averaged. The error bars show 1 standard deviation over five simulations.

Figure 9B shows a comparison between the accuracy of SNNs using the on-chip and off-chip training scheme by taking pulse-to-pulse variation into account. When an update pulse is applied to a synaptic device, a Gaussian distribution function is used to indicate fluctuations in weight updates. The variation is applied to the on-chip training SNN system whenever an update pulse is applied. On the other hand, the variation affects the off-chip training system only once when transferring the trained weights to the conductance of synaptic devices in SNNs. As shown in Figure 9B, the accuracy significantly decreases when a large conductance variation is applied to the synapses in the off-chip training SNN system. However, even if σ/μ increases to 2, the accuracy of the on-chip training SNN system is maintained (accuracy loss of 0.2% at σ/μ = 2).

We also evaluate the effects of device-to-device variation on the SNNs. Synaptic devices in the array can have various characteristics for one non-linearity factor. We assume that the non-linearity factor of synaptic devices in the array follows a Gaussian distribution, and the accuracy of SNNs is evaluated with respect to the degree of variation. As a result of applying the device-to-device variation, the synaptic device array has various conductance responses with different non-linearity factors. However, the on-chip training SNN systems also maintain their accuracy, but the accuracy of off-chip training SNN systems decreases as the degree of device-to-device variation increases, as shown in Figure 9C.

Lastly, we investigate the effect of the stuck-at-off ratio on the accuracy of SNNs. The stuck-at-off ratio is defined as the ratio of the number of stuck-at-off devices to the total number of devices in the array. Note that the number of devices with a conductance of 0 increases as the stuck-at-off ratio increases, and the stuck devices cannot be updated. As shown in Figure 9D, the accuracy of on-chip training SNNs decreases as the stuck-at-off ratio increases. A device pair represents a weight in SNNs, and both devices in the pair are updated when the corresponding weight is updated. Therefore, the weight updates are always performed using both near-linear LTP and LTD curves, which can mitigate the abrupt conductance change in the highly non-linear LTD curve of the GSDs. However, if one device in the pair is stuck-at-off with respect to all training iterations, the abrupt changes of stuck devices cannot be mitigated and degrade the performance of SNNs, even if the on-chip training scheme is adopted. When the SNNs adopt the off-chip training scheme, the accuracy of SNNs also degrades as the ratio increases, and the degree of accuracy loss is more severe than in the case of adopting the on-chip training scheme.

## Discussion

In this work, we proposed an on-chip training scheme suitable for hardware-based SNNs using analog synaptic devices. This scheme requires 2 bits of memory per neuron to update a weight: 1 bit for storing the spike event of the neuron at the last time step and the other for storing the derivative of the neuron’s activation function. Since the input of the first layer is converted to a Poisson-distributed spike train, the probability of generating a spike at each time step is determined by the activated value of the neuron. The stochastic 1-bit spike event of an I&F neuron helps the system achieve high accuracy while using the minimum memory. In addition, we evaluated the performance of the proposed training scheme in classifying N-MNIST data that cannot be represented as Poisson-distributed spike trains. As shown in Supplementary Table S1, the on-chip training SNN system achieved 97.64% accuracy with real spike data from event-based sensors (N-MNIST data) and still has the advantages of low power consumption and hardware efficiency.

As a synaptic device, we fabricated a gated Schottky diode (GSD), which has saturated current with respect to the input voltage. Even if a noisy input voltage is applied to the GSD, the weight represented by the GSD is stable because almost constant saturation current is maintained. When the on-chip training SNN system uses GSDs as synaptic devices, the array of GSDs can be updated and inhibited in parallel operation, which greatly boosts the training speed of the SNN system. In addition, the energy consumption per spike in a GSD is about 30 fJ (∼1 nA current at 3 V amplitude and 10 μs pulse width), so the on-chip training SNN system is estimated to operate at very low power consumption.

The on-chip training SNN system was verified with fully connected neural networks for MNIST data classification. The accuracy of SNNs (784-256-10) using the on-chip training scheme achieved 97.83% with *T* of 20, compared to an accuracy of 98.04% when ANN-to-SNN conversion was used with the same network. Since we did not use regularization methods such as dropout (Srivastava et al., 2014) or L2 regularization, training curves with a large λ_UP_ in deep networks can show variance, and the accuracy of deep networks can decrease. In this case, increasing *T* is a way to recover accuracy, because the activated and delta values of the neuron are more precisely represented by increased *T*. However, increasing *T* can be a burden on the overall system because the forward-propagation process is repeated *T* times in on-chip training SNNs. Increasing the batch size of the training process is also a way to enhance the accuracy of deep networks by averaging stochastic spike events of neurons within a single batch training. We confirmed that the accuracy of deep networks with increased batch size (98.44%) is very close to that of conventional ANNs (98.54%). In addition, the accuracy of deep networks can be improved by controlling the λ_UP_, which is used as the learning rate of conventional ANNs. Since the proposed on-chip training scheme uses a 1-bit spike event at the last time step, the weight updates are calculated less precisely compared to the conventional backpropagation algorithm. Therefore, setting a small λ_UP_ allows deep networks to achieve high accuracy.

We investigated the effect of the non-ideal characteristics of synaptic devices on the performance of on-chip training SNNs. Digital SNN systems seem to alleviate the influence of the non-ideal characteristics of synaptic devices (Pani et al., 2017; Yang et al., 2018; Yang et al., 2020), but analog SNN systems can be affected by such synaptic characteristics. Therefore, their influence needs to be considered when evaluating the performance of analog SNN systems. In this work, the non-linearity and asymmetry of devices affected the performance of SNNs, but high accuracy was still achieved even in the extreme case. Since the width of pulses to be applied to synaptic devices is obtained in proportion to the delta value, degradation due to non-linear weight update is mitigated in this training scheme. Compared with conventional on-chip training algorithms that use the number of pulses to be applied to update the weights, this scheme has the advantage of continuously and accurately updating the conductance of synaptic devices. As a result, this training system allows the conductance of analog synaptic devices with continuous characteristics to be reflected in the training process, thereby improving the accuracy of SNNs with non-linear synaptic devices.

Furthermore, the effects of three types of device variations on the performance of SNNs were evaluated with respect to the degree of the variation when the GSDs are used as synaptic devices: pulse-to-pulse variation, device-to-device variation, and the stuck-at-off device ratio. Since on-chip training SNNs can mitigate the impact of variation on the system performance, the accuracies of on-chip training SNN systems with GSDs are slightly affected by the pulse-to-pulse variation and device-to-device variation. In contrast, if one of the pairs of devices is stuck-at-off, non-linear weight updates by the LTD curve of one GSD device have a significant impact on the training process and degrade the performance of on-chip training SNNs. However, since GSDs are fabricated with reliable CMOS processes, the stuck-at-off ratio in the GSD array is expected to be negligibly small.

The main challenge of the proposed on-chip training scheme for SNNs is realizing the performance of convolutional neural networks (CNNs) or recurrent neural networks (RNNs). To achieve this, weight sharing in the CNN structure should be implemented in SNN systems with low power consumption (Bartunov et al., 2018). Although the max-pooling layer and softmax layer in CNNs can be implemented in SNNs (Rueckauer et al., 2017), the batch normalization layer, which significantly improves the performance of CNNs, should be implemented in hardware-based SNNs while updating parameters during training iterations. In addition, the long short-term memory (LSTM) layer in RNNs should be implemented in the form of SNNs without much memory usage. If the conditions mentioned above are met, the proposed on-chip training scheme is expected to achieve state-of-the-art performance for hardware-based SNNs with low power consumption and high-speed parallel operation.

## Data Availability Statement

The datasets generated for this study are available on request to the corresponding author.

## Author Contributions

DK, SL, and J-HB conceived and designed the experiments. DK, SL, and HK performed the simulation for MNIST classification. DK, SL, J-HB, S-TL, HK, Y-TS, JK, and J-HL performed the theoretical analyses. DK and Y-TS measured the device characteristics. DK, SO, KY, B-GP, and J-HL wrote the manuscript. All of the authors discussed the results and commented on the manuscript.

## Conflict of Interest

The authors declare that the research was conducted in the absence of any commercial or financial relationships that could be construed as a potential conflict of interest.
