# Supplementary material for: On-Chip Training Spiking Neural Networks Using Approximated Backpropagation With Analog Synaptic Devices
Source: Front Neurosci. 2020 Jul 7;14:423. doi: 10.3389/fnins.2020.00423 (PMC7358558; doi:10.3389/fnins.2020.00423)
Supplement: Supplementary file 1 [file Data_Sheet_1.PDF]

Models	Supervision	Training method	Batch size	Network	Accuracy
Cohen et al., 2016	Supervised	BP-based	-	Spiking MLP	92.87%
Lee et al., 2016	Supervised	Adam	1	Spiking MLP	98.74%
Neil et al., 2016	Supervised	Adam	-	Spiking CNN (pre-training)	95.72%
				Non-spiking CNN	98.30%
Wu et al., 2018	Supervised	Adam	100	Spiking MLP	98.78%
This work	Supervised	BP-based	100	Spiking MLP	97.64%

Supplementary Table 1. Performance comparison of proposed and conventional schemes for N-MNIST classification in SNNs. In this work, the network is fully connected and its size is (34×34×2)-1500-10. The event stream of each image sample has a 300 ms period (100 ms×3 saccades). In the simulation of the network using the proposed scheme, we set T to 300 ms with a time step of 1 ms. The parameters in the network are updated every 10 ms, and softmax function is used in the last layer. As a result of training, the accuracy in this work is 97.64%. Compared to other training schemes for SNNs, the proposed scheme shows slightly lower accuracy. Since the input signal of N-MNIST data is not a Poisson-distributed spike train, storing a 1-bit spike event is less meaningful than when the spikes for the input signal are Poisson-distributed. In addition, we do not use Adam optimizer which is known as a popular and powerful optimizer, since implementing it in hardware is expected to result in high circuit complexity, large area occupancy and high power consumption. However, even given spike data from an event-based sensor, the proposed scheme for SNNs achieves comparable accuracy while using minimal memory for storage of a 1-bit spike event per neuron, and still has the advantages of low power consumption and hardware efficiency.

References

Cohen, G. K., Orchard, G., Leng, S. H., Tapson, J., Benosman, R. B., and Schaik, A. V. (2016). Skimming digits: neuromorphic classification of spike-encoded images. *Front. Neurosci.* 10:184. doi: 10.3389/fnins.2016.00184

Lee, J.-H., Delbruck, T. and Pfeiffer, M. (2016). Training Deep Spiking Neural Networks Using Backpropagation. *Front. Neurosci*. 10:508. doi: 10.3389/fnins.2016.00508

Neil, D., and Liu, S. C. (2016). Effective sensor fusion with event-based sensors and deep network architectures," in IEEE International Symposium on Circuits and Systems. *ed O. René Levesque (Montréal, QC).*

Wu, Y., Deng, L., Li, G., Zhu, J, and Shi, L. (2018). Spatio-Temporal Backpropagation for Training High-Performance Spiking Neural Networks. *Front. Neurosci.* 12:331. doi: 10.3389/fnins.2018.00331
