# Supplementary figures and images for: On-Chip Training Spiking Neural Networks Using Approximated Backpropagation With Analog Synaptic Devices
Source: Front Neurosci. 2020 Jul 7;14:423. doi: 10.3389/fnins.2020.00423 (PMC7358558; doi:10.3389/fnins.2020.00423)

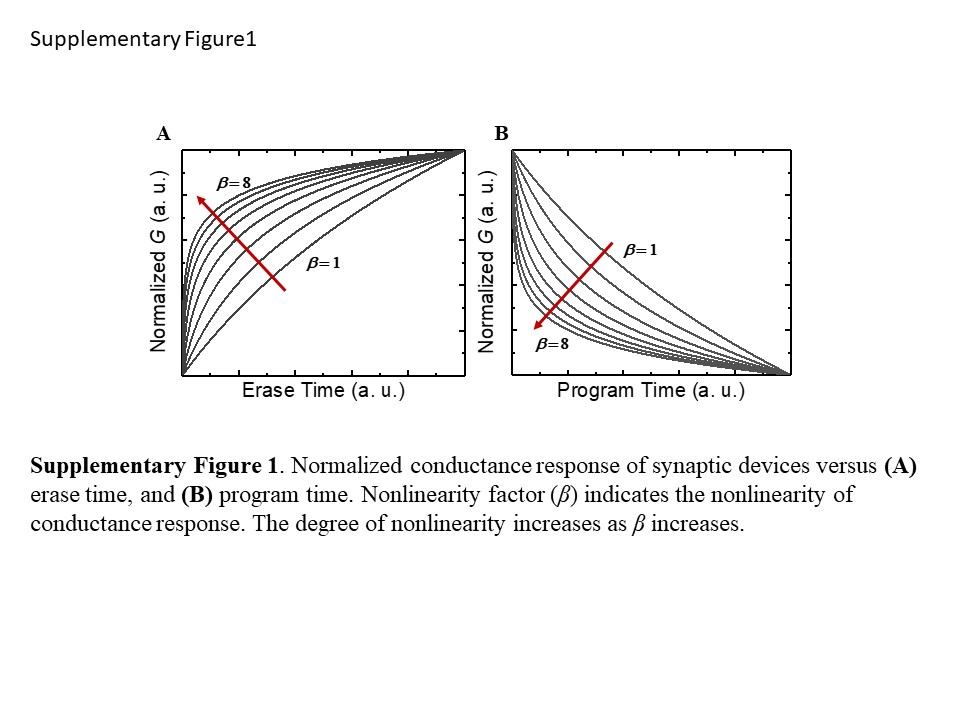

Supplement: Supplementary file 2 [file Image_1.JPEG]
